# Of Dark Arts and Dictators—Why the Russian Constitution Matters: The Constitutional Dark Arts by William Partlett^†^

**DOI:** 10.1093/ojls/gqag015

**Published:** 2026-05-05

**Authors:** Alasdair McCallum

**Keywords:** comparative constitutionalism, political theory, constitutional law

## Abstract

Analysis of authoritarian constitutions is a growing but still relatively underdeveloped body of literature. The traditional explanation is that these constitutions are no more than ‘shams’ or ‘fig leaves’. William Partlett’s book *Why the Russian Constitution Matters: The Constitutional Dark Arts* challenges this, placing the Russian Constitution at the centre of a phenomenon Partlett terms the ‘constitutional dark arts’, a practice combining a hyper-centralisation of power in the elected president with the claim to be upholding rights and democracy. In doing so, it offers both a new constitutional history of Russia and a new generalist paradigm of the ‘constitutional dark arts’ which can be applied in both authoritarian states and democratic states undergoing backsliding and creeping autocratisation. Partlett’s book answers the question of whether the Russian Constitution matters in the affirmative, and will be of interest to scholars of comparative constitutionalism, democratic decline, and Russian and post-Soviet studies more broadly.

## 1. Introduction

William Partlett’s book *Why the Russian Constitution Matters: The Constitutional Dark Arts* (Bloomsbury 2024) makes the argument that the Russian Federation is not a mere fig leaf or ‘sham’, but instead establishes a coherent basis of rational-legal authority that has allowed the Putin regime to survive for two and a half decades. It argues that the Russian Constitution does so through the use of the ‘constitutional dark arts’, which elevate the president above other branches of power under the 1993 Constitution while claiming to protect human rights and democracy. Partlett makes the argument that this is not an abuse of the Russian constitutional order established in 1993, but rather serves as a direct consequence of its hyper-centralisation of power under the president. This review article aims to evaluate Partlett’s work and synthesise research from the fields of comparative constitutional law, political science and history to make a valuable contribution to studies of authoritarian legalism and constitutionalism. The article will first turn to the central thesis of Partlett’s book, before addressing the theory and methodology utilised in the book. It will then turn to the practical application of the theory in terms of the temporal and historical dimensions of the thesis. I will also refer to theoretical and comparative material that could have enriched the book’s argument.

## 2. The Central Thesis

Partlett’s book turns quickly to advancing and defending the central claim of the book. He relies on the Russian example to outline a generalist paradigm of the constitutional dark arts. Partlett outlines the constitutional dark arts as follows:

Russia’s constitutional drafters (and their supporters in the West) in the early 1990s relied on a neglected constitutional practice that I call the ‘constitutional dark arts’. This practice—which has grown in influence globally since the 1980s—guarantees democratic principles and individual rights within a highly centralised constitutional structure. This creates the illusion of democracy while in reality undermining both democracy and rights-protecting governance.^1^

These practices are ‘dark arts’ because they are illusory, duplicitous and superficially appealing, and create a system in which power is concentrated in one elected leader. The constitutional dark arts rest on two premises. The first is popular accountability, which draws from the ‘anti-politics’ of the centralised state tradition. This frequently assumes that there is a unified public will which is capable of holding the chief executive to account, often formalised in constitutional structures which state that ‘the people’ are the sole source of authority in their foundational provisions and claiming the president embodies the will of the people in subsequent provisions.^2^ Proponents of the constitutional dark arts argue that an elected leader, even one with few checks on their power, can serve as the unmediated and legitimate representative of the people as a whole.^3^ The second defence is that the constitutional dark arts ensure legal accountability—that ‘centralised power ensures stable governance and a unified system of legality, allow[ing] courts to hold the state legally accountable for any democratic or human rights violations’.^4^ International human rights are acknowledged, but frequently not respected; the judiciary are supposed to serve as a kind of ‘insurance’^5^ to protect rights and restrain state power, but are frequently incapable of doing so. Partlett’s thesis is that the constitutional dark arts are intimately connected with several specific modes of organising power: the centralised state tradition and the governing model of crown-presidentialism. Other key themes that tie the book together are the notions of guardian-style constitutional powers and management-style presidential powers, and the ‘anti-politics’ of the Russian state tradition.

Partlett advances what he terms a ‘structural-normative approach’ to addressing the constitutional dark arts. This is an approach that considers not just the bare content of the Russian Constitution, but also the normative rules that underpin this formal content. He argues that the organisation of power in the Russian Constitution serves as the foundation of ‘a broader normative system for how politics should be organised’.^6^ In foregrounding the normative aspects of the Russian Constitution, Partlett’s ideas reflect Barber’s notion of the ‘constitutional ideology’^7^—that constitutions are not simply legal documents but ‘ideological devices’—to build on the standard rules-based interpretation of constitutional design.

Partlett has written an extensive body of work on the Russian Constitution that places it within a comparative legal and historical framework. His previous contributions include creating a methodological framework for identifying ‘crown-presidential’^8^ systems, including Russia, which is an idea he returns to frequently in *Why the Russian Constitution Matters*. This is in addition to his work on Russia’s ‘non-transformative’ founding period from 1991 to 1993, prior to the promulgation of the 1993 Constitution^9^ and the failure of the genuinely semi-presidential system established in this period,^10^ in addition to commentary on Russia’s 2020 Constitution amendments.^11^  *Why the Russian Constitution Matters* ties these different threads together into the concept of the constitutional dark arts.

Partlett’s book is divided into 10 body chapters. At its outset, the book offers a theoretical framework for evaluating the constitutional dark arts, before addressing Russia’s constitutional development over several historical periods spanning the 1970s dissident Democratic Constitutional Movement to the present. Finally, the book offers a number of lessons about constraining the constitutional dark arts and renewing democratic constitutionalism.

## 3. Re-evaluating the Core Arguments on Authoritarian Constitutions

### A. Sham Constitutions? Why Constitutions in Authoritarian States Matter

Partlett revises both generalist scholarship regarding the nature of authoritarian constitutions and scholarship on the Russian Constitution and the long shadow cast by the Russian constitutional crisis of 1993. Partlett answers the question of whether the Russian Constitution matters in the affirmative, which goes against an older line of scholarship that continues into the present day that rejects the significance of constitutions in authoritarian states (Partlett calls this line of argument the idea of ‘sham constitutions’).^12^ For example, in the 1960s Giovanni Sartori addressed the constitutions of socialist states like the Soviet Union and argued that these were not ‘real’ constitutions, instead terming them ‘nominal’ or ‘facade’ constitutions,^13^ and further argued that constitutions are required to observe the principles of constitutionalism in order to be valid. However, this sits uneasily with a more recent line of scholarship re-evaluating the importance of authoritarian constitutions, encompassing Partlett’s work and that of others who reconsidered the value of constitutions ranging from the 1936 USSR Constitution^14^ to the Chinese Constitution of 1954,^15^ that of Myanmar in 2008^16^ and the 1997, 2007 and 2017 Thai constitutions.^17^

The conventional explanation is that constitutions in authoritarian states do not matter.

Smirnova and Thornhill write:

One common presumption in research on this topic … is that the Russian constitution is located in a sphere that is far removed from meaningful reality, and its importance as a fixed legal order is overshadowed by the informal norms and informal institutions that shape factual interactions in society and in government.^18^

However, scholarship has demonstrated that constitutions in authoritarian systems do have significance, even if not necessarily in the same way as democratic constitutions. Ginsburg and Beeman demonstrate that authoritarian constitutions provide additional functions such as regime stabilisation and easing transitions of power.^19^ Landau warns of ignoring formal constitutional rules in authoritarian regimes, noting that they define critical functions such as tenure, power over institutions and other variables,^20^ while Hale rejects this binary of ‘formal’ and ‘informal’ politics, citing Ukraine’s divided-executive constitution as facilitating democratisation despite the ongoing importance of informal practices in Ukraine, especially from 2005 to 2010.^21^ Authoritarian constitutions are not mere ‘shams’ and do have real significance.

### B. The Shadow of Yeltsin and 1993

Much literature on the Russian Constitution grounds its arguments in the claim that Russia’s Constitution had a democratic beginning under Yeltsin and subsequently fell into misuse and abuse under Putin. The 1993 Constitution still attracts the claim that it was ‘a flawed but forward-looking document’ that was only ‘stripped of its potential’ in the controversial 2020 referendum.^22^ Thus, Russia is a ‘post-democracy’^23^—it had a democratic foundation that was destroyed by Putin’s authoritarian tendencies and shifted from using formal constitutional mechanisms of power to informal practices removed from the Constitution under Putin.

As previously indicated, Partlett’s work takes the revisionist stance that Russia’s authoritarian turn under Putin is not an aberration born out of a departure from constitutional design, but rather a continuation of principles that were present in the 1993 Constitution from the outset.^24^ Putin’s rise to the presidency is often seen as a break in terms of constitutionalism and the rule of law in Russia. However, Partlett’s book instead finds continuity from the promulgation of the 1993 Constitution to the present. This was not a case of what Claus calls ‘constitutional capture’ by which an authoritarian leader takes a democratic constitution and destroys it from the inside, but rather an established reality from the outset.^25^ Bypassing the usual horizontal divisions of power, Yeltsin made ample and repeated use of his extensive executive powers, most notably the power to veto legislation and issue normative decrees, to override the legislature in the 1990s, well before the age of Putin. In the lead-up to the 1996 presidential election, when it was feared that a Communist victory could undo much of the process of privatisation, Yeltsin invoked his veto power 180 times in three months.^26^ The pattern of decree making did not decline, nor did the number of decrees promulgated, but the ‘patchwork’^27^ approach of the Yeltsin years declined as the State Duma passed a much larger volume of laws than in the Yeltsin period.^28^ Putin drew on the vast sovereign authority given to the Russian president in the latter articles of the Constitution and did not unilaterally assert these powers *ex novo*, but simply amplified the powers present in the Yeltsin period—Putin built on new offices and powers, but largely did not create them anew. For example, Putin took offices like the Presidential Administration, which had been established by Yeltsin by decree in the early 1990s,^29^ but gave it vastly increased additional powers.^30^

The book makes the case that Russia’s failure to make a successful democratic transition was not the product of under-institutionalisation and the underdevelopment of constitutional norms. The 1993 Constitution established rational-legal authority in Russia with long-lasting implications but, rather than contribute to a balanced constitutional order, served instead to create a rational-legal basis for personalised power. In making this argument, Partlett’s book offers a new constitutional history of the Russian Federation that differs from much other scholarship, much of it outdated, on the Russian Constitution.

## 4. Theory and Methodology

### A. Anti-politics and Political Ordering: The Centralised and Balanced State Traditions

After outlining the ‘constitutional dark arts’, Partlett immediately turns to outlining what are the other central theoretical notions of his book. Partlett outlines the ‘centralised state tradition’, observing:

Since the 1980s, however, constitutional centralisation has been increasingly justified as a better way to achieve democracy and individual rights. This deceptive claim envisions an anti-political constitutional system where an elected sovereign leader oversees a state limited by judicially enforced rights and democracy guarantees. This kind of Constitution is then justified as the best of both worlds: able to create a strong, unified state that is also democratic and rights protecting.^31^

He writes: ‘This tradition values a kind of “anti-politics” that condemns political pluralism and compromise. It holds that good political ordering requires a constitution that encourages unity by subsuming or eliminating internal rivals in favour of a central power’.^32^

The centralised state tradition does not frame itself as undemocratic. The sovereign’s legitimacy is populist and plebiscitary—as long as they are able to keep winning elections, they have the right to remain at the apex of rational-legal authority without meaningful constraints on their power. Instead of drawing on a pluralistic constitutional order with a president restrained by horizontal checks and balances, constitutions like the Russian Constitution appeal to the centralised state tradition for democratic legitimacy. Constitutions of authoritarian countries are often created by states amidst crises and appeal to a sense of urgency to justify invoking the centralised state tradition. The Russian Constitution of 1993, enacted in the wake of Boris Yeltsin’s unconstitutional seizure of power from the elected legislature, is a prime example.

The centralised state tradition of constitutionalism does not always lead to the end of democracy when this state of emergency is temporary: the formation of the French Fifth Republic in 1958 serves as a counterexample of when democracy can survive a state of emergency. The suspension of the Indian Constitution in the 1975–77 constitutional ‘emergency’ in India under Prime Minister Indira Gandhi serves as a cautionary tale, as Gandhi restored democracy after 21 months and was held accountable by voters, who voted her out of office. The centralised state tradition becomes dangerous when the extraordinary powers awarded to the sovereign are routinised. Russian ‘crown-presidentialism’ essentially routinises this state of exception and makes the president akin to an elected monarch.^33^

Concerningly, this centralised state tradition is gaining ground in Western democracies, where popularly elected leaders increasingly base their appeal on a claimed unmediated link between themselves and the people. In Hungary, for example, Prime Minister Viktor Orbán has also appealed to the centralised state tradition to consolidate his power. Perhaps the most familiar example is Donald Trump, who has repeatedly derided other branches of government, frequently declared states of emergency in American cities and threatened the judiciary and city governments that he will act unilaterally to overrule them if they defy his wishes. The normalisation and routinisation of the centralised state tradition is far less remote than it may appear to us in the West and makes vigilance against democratic decay all the more important.

### B. The Anti-political Tradition

After expanding these theoretical motions, Partlett demonstrates how the constitutional dark arts have informed Russia’s constitutional history through a series of historical chapters that periodise Russia’s constitutional development. Partlett links this anti-political tradition back to Hobbes, the Prussian and imperial Japanese constitutions and those of Communist states, which, as Partlett states, all ‘value a form of anti-politics in which the sovereign officeholder ensures harmony and unity’.^34^ The second chapter^35^ covers Russia’s democratic constitutional movement and constitutional thinking in Russia from the Sakharov dissident tradition up to Boris Yeltsin’s constitutional draft of 1993. While expressing respect for Sakharov and the dissident movement in moving beyond the rigid Soviet Constitution, Partlett observes that their constitutional thinking was largely grounded in a centralised and anti-political tradition, rejecting institutions like political parties while affirming an approach grounded in international law and rights. Sakharov and his supporters ‘deliberately refused a political program that sought to fundamentally change the institutional position of the communist party or introduce political pluralism’ but instead ‘appealed to the universal, moral and international principles of individual rights’,^36^ which would prove to be an influence on the 1993 Constitution.

### C. Crown-Presidentialism: The Central Role of the Presidency

The Russian political system is often studied in terms of the huge powers it lends the office of the presidency. The power of Russia’s presidency under the 1993 Constitution is immense, and Russia’s constitutional system has been referred to in such terms as a ‘superpresidency’, ‘superpresidential’ or ‘superpresidentialism’.^37^ What Partlett’s book sets out to do is provide an analytical framework—the constitutional dark arts—through which this immense power can be dissected and applied comparatively to other systems. The presidency towers over the other constitutional bodies in Partlett’s book, and thus this book is as much about why the Russian presidency matters as why the Russian Constitution matters. There have been several monographs evaluating the strength of the presidency and questioning the importance of the presidency and its role in Russia.^38^ Since the Russian invasion of Ukraine in 2022, the essential role of the presidency has become beyond doubt, and thus the periodisation seen in the book along the lines of presidential terms is not a straitjacket but simply a reflection of a reality in which a single office holder possesses an immense degree of power. Contemporary scholarship referred to the Russian Constitution as an ‘Airbus’,^39^ put together from different components of several democratic constitutions. However, this improvisational ‘Airbus’ became something far more dangerous. Partlett quotes Kim Lane Scheppele as describing Russia as a ‘Frankenstate’,

where the ‘inter-action effects’ of ‘perfectly reasonable pieces’ ultimately leads to the creation of a monster … a presidential office that has the power to dominate both formal and informal political ordering. Simultaneously reflecting and bolstering the centralised state tradition, crown-presidentialism creates a kind of permanent state of exception with a sovereign President.^40^

Partlett argues that the role of the Russian president has gone through several stages which can be easily periodised. In the third chapter, which covers the ‘foundation of the constitutional dark arts in Russia’, Partlett establishes the critical role of former Russian President Boris Yeltsin and the 1993 Constitution in establishing the constitutional dark arts in Russia.^41^ Chapter 4^42^ covers the main part of Yeltsin’s presidency from 1994 to 1999—the period that Partlett terms the ‘personal president’. Chapter 5^43^ covers Putin’s first two terms, from 2000 to 2008, which Partlett terms the ‘managerial president’, while Chapter 6^44^ covers 2008 to 2012, which Partlett terms the ‘constrained president’. Partlett argues that the replacement of Vladimir Putin by Dmitry Medvedev as president from 2008 to 2012, often termed the *rokirovka* (castling) in Russian political parlance, was not merely a fig leaf with Medvedev acting as Putin’s pawn. He notes Medvedev’s more conciliatory approach to foreign policy, especially Russia’s abstention from the 2011 United Nations Security Council vote authorising military action in Libya, which Putin criticised only to back down, and the decision to fire Moscow’s powerful mayor, Yuri Luzhkov, apparently without Putin’s input.^45^

Putin returned as president in 2012, after Medvedev had served a single term. Chapter 7 covers 2012 to the present and argues that in this period, Russia made the change from a ‘constrained’ presidential order under the Medvedev/Putin tandem to an ‘imperial’ presidency as an immense degree of power became concentrated in Putin’s hands.

### D. Guardian and Management Powers of the Presidency

The book differentiates between the guardian (French-style) and management (US-style) elements of Russian presidential power under the 1993 Constitution. The French-style guardian powers intrude into the legislative and judicial branches of government in line with the president’s role in Russia as the ‘guarantor’ (*garant*) of the constitution and include the Russian president’s power to dissolve the lower house of parliament, determine foreign policy, act as commander-in-chief of the armed forces and, since 2020, ensure that all bodies function and interact as a single system of public power.^46^ The American-style management powers include extensive powers over the executive branch, such as the power to dismiss the prime minister and ministries/government, to submit bills to parliament, to unilaterally form the presidential administration and to organise executive branch institutions. The book observes a flaw in how the Russian Constitution is often viewed by scholars: it is often described as a semi-presidential system that follows the French model,^47^ but Partlett notes that the extreme guardian powers granted to the president in Russia mean that this is not the case. The Russian Constitutional Court ruled in 1998 that the president could nominate the same prime ministerial appointment who the legislature had previously rejected three times,^48^ only to dissolve the legislature and appoint that nominee, meaning that the Russian constitutional order is not truly semi-presidential because the president is not truly accountable to the legislature.^49^ The immense powers concentrated in the presidency would increasingly be abused by Yeltsin’s successor Vladimir Putin, culminating in the 2020 constitutional referendum and its ‘nullification’ (*obnulenie*) of Russia’s constitutional two-term limit on presidential terms, allowing Putin to potentially remain in power until 2036.

## 5. Theory and Application: Temporal/Historical Dimensions of the Thesis

The temporal dimension of Partlett’s book focuses on the changes in Russian constitutional tradition from the 1970s to the present. Partlett takes a historical perspective on the centralised state tradition, noting that it has operated in various ways throughout recorded history. Partlett traces the history of the centralised state tradition through the Prussian Constitution of 1850 and the Constitution of the Japanese Empire of 1889.^50^ He notes that in the Leninist constitutions of Communist countries, governments reject the separation of powers in favour of the principle of ‘democratic centralism’,^51^ subjecting all legal authority to the will of the ruling party. However, Partlett observes that since the 1980s the tendency has changed to a new, universalised constitutional discourse about democracy, rights and the separation of powers.^52^ While constitutions even in authoritarian states often incorporate democratic rhetoric, the recent trend among authoritarian rulers has been to incorporate rights and international law discourse into a system that concentrates immense power in the executive presidency, with this power tending to grow over time.

Given the temporal focus of the book, it might have been appropriate to see some further engagement with previous Soviet constitutions. Particularly given Partlett’s scepticism of rights as the basis for protection of democratic freedoms under the Constitution, Partlett could have addressed the 1936 USSR Constitution, which extensively and ineffectually refers to individual rights, in more detail. Partlett does briefly address challenges to the rights provisions of the 1936 Soviet Constitution,^53^ which extensively discussed the rights and duties from the drafting process through to the promulgation of the Constitution itself,^54^ but additional engagement with the 1977 Soviet Constitution and the 1978 RSFSR Constitution could also have contributed to the historical narrative that Partlett weaves throughout this book.

### A. The 2020 Constitutional Amendments

In 2020, a raft of changes to the Russian Constitution were passed and affirmed by a non-binding referendum. Some scholarship appreciates the significance of the 2020 constitutional amendments in terms of their sheer length—they change over one-third of the Constitution, with Bui Ngoc Son describing them as Russia’s ‘big bang constitutional amendments’.^55^ Nonetheless, Partlett states that ‘none [of the amendments] made any serious functional changes to the overall constitutional system’.^56^ The amendments show a superficial commitment to reducing the management power of the presidency, but ultimately increase both the president’s guardian and management powers.^57^

There is considerable scholarship on the 2020 amendments and the values that they signal,^^58^^ and considerable literature on ideology and constitutions more generally.^59^ Namely, the 2020 constitutional amendments promulgate what Partlett has previously called a ‘protectionist’ constitution, dedicated to ensuring social and economic welfare and social conservatism.^60^ This signalling of values is a familiar part of authoritarian constitutions,^61^ but the 2020 amendments signal Russian exceptionalism rather than the universalist signalling preferred by Yeltsin.

In 2020, the separation of powers was abrogated in favour of a new concept of ‘public power’ (*publich’naia vlast’*), which replaced what one expert called the now ‘outdated’ (*ustarevshee*) separation of powers enshrined under section 10 of the Constitution.^62^ The system of public power contemplated in the 2020 constitutional amendments appears to be a complete embrace of Agamben’s doctrine that the state of exception: ‘the provisional abolition of the distinction among legislative, executive, and judicial powers—here shows its tendency to become a lasting practice of government’.^63^ Before 2020, the problem was not the lack of a separation of powers, but rather the fact that there is an institution—the presidency—that sits above all of these. Russia ‘had separation of powers without any checks on presidential authority’.^64^ While a clear constitutional distinction is made between the different powers, there are few meaningful checks and balances, and with the sovereign (the president) sitting above the different levers of power, the state of exception is preserved by the inability of the elected sovereign to be checked by any of the other, ‘lower’ branches.

## 6. Application and Doctrine: Comparative Dimensions of the Thesis

The comparative dimension of the book places Russia in the context of other ‘crown-presidential’ systems worldwide. Constitutions are products of their histories and cultural contexts, and an approach grounded more explicitly in area studies and the diffusion process of ‘authoritarian learning’^65^ might provide for a better model for this work. There continues to be work based around geographic ‘families’ of constitutions^66^ and Partlett could have adopted this approach considering the direct impact of Russia on other authoritarian systems. Mills and Joyce^67^ coined the term ‘norm transmission’, which was adapted by Roberts to comment on the ‘authoritarian norm diffusion’ of Russia’s political norms into adjacent post-Communist states,^68^ especially given Russia’s role as regional hegemon.^69^ This has been observed in the context of normative legal acts, with Kazakhstan ‘copying’^70^ Russia’s notorious ‘Foreign Agents’ Law’ and ‘homosexual propaganda’ law.^71^ This process of copying and lending from Russian law can be extended to constitutional provision provisions. Indeed, one scholar has written on a distinctly ‘Eurasian’ style of constitutional design, with the Russian Constitution serving as a ‘regional template’.^72^ Partlett could have enhanced this section by more specifically grounding his argument not just in terms of analogy, but also in terms of Russia’s direct diffusion of norms and influence on other, regional ‘crown-presidential’ systems. While the global comparisons are welcome, given Partlett’s description of the Russian president as an ‘elected monarch’,^73^ he could have turned to some other systems which feature hybrids of presidential and monarchical rule that place the monarch ‘above’ other branches of government and have also undergone transitions in terms of the apportionment of powers, including Morocco^74^ and Thailand.^75^ Both the Moroccan and Thai constitutions give extraordinary powers to the monarch within a system of checks and balances and apportionment of powers. Partlett does refer to the Zimbabwean^76^ and South Sudanese^77^ constitutions, which also place few limits on the power of the elected president. These constitutions claim that the elected president has an unmediated relationship with the people, allowing authoritarian leaders to superficially adhere to democratic norms and escape international scrutiny while nonetheless consolidating personalist authority.

Other examples may be more familiar to scholars. Partlett follows how in Turkey, Recep Erdoğan made drastic changes to the Turkish Constitution in the wake of an attempted military coup in 2016, changing the system of government from a parliamentary system into a presidential one that consolidated immense powers in himself.^78^ As in Russia in 2020, Turkey turned to a constitutional referendum as a legitimation mechanism,^79^ and Erdoğan has repeatedly used his narrow victories in presidential elections to claim a plebiscitary mandate for his rule. Both Russia and Turkey have utilised the constitutional dark arts by turning to constitutional referendums to legitimise authoritarian consolidation, a superficially democratic measure that ultimately aids in the centralisation of power. This centralised state tradition has also re-emerged in formerly apparently stable democracies, such as Hungary under Viktor Orbán, where Orbán has turned to both referendums and multiple non-binding plebiscites on sensitive issues (‘national consultations’) to entrench his rule while superficially upholding democratic norms.^80^

According to Partlett, it is not a complete rejection of the centralised state tradition but rather a kind of a pluralism of institutions that is needed. He observes that the balanced state tradition can have its own faults—namely, it is too decentralised and private actors have too much power.^81^ Centralised sovereign authority ‘can yield important short-term stability in times of crisis or emergency’,^82^ but over time it creates personalisation and lack of accountability. However, he states that the Russian Constitution need not be replaced, writing that chapters 4, 5 and 6, which organise power, and article 10, which is related to the powers of the presidency, the constitution would require only minor revisions.^83^ Partlett does not endorse a specific vision of the Russian state, but he does suggest that a ‘semi-parliamentary’ system along Australian lines could be adopted, while referring to, though not specifically endorsing, the ‘parliamentary republic’ envisaged by opposition figures such as Alexei Navalny.^84^

## 7. Application and Doctrine: Scepticism of Rights and Courts

Partlett also highlights how the judiciary has been an inadequate protector of individual and constitutional rights in Russia. As Partlett observes, without detailed structural provisions in the constitution, in systems like Russia with weak courts, abstract constitutional provisions guaranteeing rights are open to different interpretations, while constitutional rules are often ‘implemented and shaped by political actors’.^85^ Although Chief Justice Valery Zorkin was one of four Constitutional Court justices to declare Boris Yeltsin’s power grab in 1993 to be illegal^86^ and was subsequently removed from the position of chief justice, the constitutional court was restructured in 1995 and since then has largely interpreted the constitution in terms of broad presidential powers.^87^ Zorkin subsequently returned as court president in 2003 and has largely become an agent of presidential power, failing to defend the limits set by the Constitution in favour of approving Putin’s personal decisions.^88^ While Russia’s Constitutional Court continues to uphold individual rights in certain cases,^89^ especially in areas related to social welfare rights,^90^ its experience tells us that we should heed Partlett’s warning that the presence of a formally strong Constitutional Court is unlikely to be sufficient to uphold individual rights. Khalikova has done a quantitative analysis of Constitutional Court cases and notes how the percentage of judgments with dissenting judgments has declined precipitously since the 1990s, and that the Constitutional Court largely steers clear of rulings that might trouble the executive.^91^ Chief Justice Zorkin has repeatedly defended the increasingly ideological trend of the Russian Constitution in his extra-curial writings,^92^ and defended the 2020 amendments as restoring ‘balance’ to the Constitution as a return to fundamental Russian values.^93^ The Constitutional Court has become increasingly disempowered over time, and since it was restored in 1995 it has largely adhered to the centralised state tradition.^94^ In systems like Russia, where the centralised state tradition presides, the executive has great power to pressure and influence the courts, and the ability of courts to check the power of the executive is unlikely to persist over time.^95^

Partlett is thus critical of the fixation with enshrining individual rights and creating an empowered Constitutional Court as a litmus test of constitutional design. He notes that enshrining an ‘anti-political approach’ that focuses on the legitimacy of a powerful popularly elected president and a court system is ‘unlikely to protect democracy and rights’^96^ when the president sits above other branches of power. This has already been noted in the context of authoritarian constitutions, with Ginsburg and Beeman observing that there is a need to move away from ‘the American fetish with judicial enforcement’^97^ and consider the alternative designs and functions of courts in authoritarian systems. Most histories of constitutional development take the Anglo-American legal tradition as their basis^98^ and transplant both the form and functions of the American Constitution into culturally and normatively very different systems to assess the legitimacy of constitutions. Landau notes that specific constitutional ‘recipes’ across countries that list institutions such as constitutional courts are often easily abused by authoritarians,^99^ while Scheppele cites the case of Hungary to bluntly conclude these indicia, which they term ‘government checklists’, ‘do not work’.^100^ Clearly, another approach is needed.

Partlett also expresses scepticism of constitutional rights as a guarantee of human rights in Russia. While the Russian Constitution lists an extensive series of individual rights (expanded in the 2020 constitutional amendments), a growing body of scholarship rejects the idea that a plethora of enumerated rights results in meaningful rights protection. Constitutional provisions that nominally uphold rights can essentially be aimed at placating the demands of a particular group^101^ while eroding democratic accountability, as an attempt to build and hold together electoral coalitions^102^ or as ‘crowd pleasers’ that nominally enshrine guaranteed nationalist and social demands.^103^ International law and democracy promotion have also consistently largely failed to constrain the office of the Russian president. The United States underinvested in both economic aid and democracy promotion in the 1990s in Russia^104^ and this trend has largely continued. Partlett observes that the ‘technical, legal’ approach favoured by international bodies is unlikely to work given the concentrated power in the hands of the president, and instead favours a structural approach which focuses on the ‘domestic organisation of political power’.^105^ The initial Russian Constitution of 1993 stresses the role of international law, which is one reason it was largely accepted by Europe, Western leaders and Russian liberals as fit for purpose.^106^ For example, the Council of Europe’s (the Venice Commission) assessment of the 1993 Russian Constitution focused on human rights provisions and did not take sufficient note of the immense powers concentrated in the presidency,^107^ and the constitution has gradually broken away from constraints on executive power. Following a decision of the Russian Constitutional Court in 2015, the ‘fundamental principles and norms of the Constitution’ have been given primacy over international law,^108^ a principle that was constitutionalised in 2020.^109^ Russia’s full-scale invasion in Ukraine in 2022 marked the final separation of Russia from the European legal system and marked a new unwillingness of the Constitutional Court to consider sensitive constitutional challenges.^110^

### A. The Final Chapters: The Russian Constitutional Order Post-Putin

The final chapters of *Why the Russian Constitution Matters* are welcome, forward-facing additions to a book that has previously been focused on history and the present. In considering the potential for a new democratic constitution for Russia, Partlett turns to a historical-cultural argument. I would make several points related to these arguments. In the eighth chapter, ‘Constitutional Law in a Post-Putin Russia’,^111^ Partlett aims to make a prognosis of how a future constitutional order in Russia might be achieved. He also makes a contentious claim rooted in a historical-cultural approach; he calls for the rejection of an ‘international approach’ and calls instead for a ‘historically rooted version of Russian democracy’.^112^ Partlett stresses that international law is often ineffective because it is seen as an outside imposition of power from the West, and that international bodies are concerned about impinging on national sovereignty.^113^ He rejects the idea that the Russian state has no liberal tradition, and looks back to the Tsarist period of the Decembrist revolt of 1825 and the Constitutional Democrats (*Kadet*) party from the late Russian Empire.^114^ However, I would contend that these examples are very old and never represented more than a minority of elite political thought in Russia. These were elite-driven traditions that often lacked a grounding in the institutional structures of the state—and, indeed, Partlett notes the problems of this approach when he discusses the flawed ‘anti-politics’ of Sakharov and the Russian constitutional movement. The book could have benefited from some additional discussions of the difficulty in amending and modernising constitutions, especially given his argument that the Russian Constitution requires only minor amendments to be fit for purpose.^115^ Examples like Chile illustrate the extraordinary difficulty of changing a constitutional order even when an old system is recognised as failing—Chile has faced two constitutional referendums to replace the Pinochet-era Constitution, and neither has succeeded.^116^ A further problem is that an approach that leans too far into cultural relativism and ‘civilisationism’ can embolden populist authoritarians and constitutional nationalists, just as Russia has stressed its unique ‘constitutional identity’ today. A balance must be struck between an ‘international approach’ and a constitutional order that highlights the ‘Russian roots’ of the new constitutional order.

The ultimate reality is that Russia may need another elite-driven ‘executive constitution’^117^ along the top-down lines established in the French Fifth Republic in 1958. The difficulty is ensuring that this is a De Gaulle constitution rather a Yeltsin one. The constitution after Putin is likely to emerge from crisis and can transcend crisis, but only if the actors involved demonstrate a normative commitment to democracy. I am concerned that the cultural-historical examples of Russian constitutionalism ultimately do not give us much hope for the creation of a constitution along these lines. However, we can console ourselves knowing that there are countries like Japan in which little liberal tradition existed, in which constitutional democracy was nonetheless established and persisted.

### B. Potential Comparative Angles

Given that Partlett dedicates two chapters to countering the constitutional dark arts and renewing democratic constitutionalism respectively, there are several examples from currently democratic states that Partlett could have identified as being highly salient to the Russian example. Given Partlett’s comparative focus, the highly analogous example of the French Fifth Republic, which was also a constitution of crisis enacted near-unilaterally amidst a period of state turmoil, but unlike Russia did not lead to a period of protracted authoritarian rule, makes a strong comparison. Partlett has even noted in a previous article that Yeltsin compared himself to De Gaulle in calling for the 1993 Constitution,^118^ as did Western journalists in rationalising Yeltsin’s actions in 1993.^119^ Given Partlett’s ‘structuralist-normative’ approach, it would have been good to see comparisons with post-Soviet systems with similar normative structures in outlining the case for a new democratic order for Russia. Partlett also accurately names several failures in protecting Russian democracy in terms of rights, courts and democracy promotion, but his book could benefit from analysis of former Soviet countries that emerged from a similar background but effectively established constitutional order and the potential lessons that could be learned for the Russian example.

One potential answer lies in the fact that some countries in transition have introduced interim constitutions and interim constitutional amendments. Grover comments that while the enactment of interim constitutions and constitutional measures is quite common historically, it has yet to receive substantial attention in the scholarship.^120^ Interim constitutional measures might have helped Russia to fill the void in legal authority and fulfil a pluralism of institutions, giving less powers to the elected president. In states where democratic norms and institutions are weak, temporary constitutional measures can clearly help in establishing a base for democracy drawn from situational experience and trial and error.

Poland’s transition to democracy is an instructive case. Wojnicki notes that interim constitutions are a longstanding practice in Poland, with interim provisional constitutions having been enacted in 1919 and 1947.^121^ These were ‘crucial moments of Polish statehood’,^122^ taking place at critical times of transition. At the time of the collapse of Communism, Poland still lacked the constitutional framework for the transition into a liberal democracy. Poland faced similar issues to Russia in 1993—political legitimacy, economic challenges and nation building—but chose to adopt an incremental form of constitution making. Amidst the breakdown of the Communist system in 1989, the Polish People’s Republic introduced constitutional changes (the July Constitution) that provided for a power-sharing arrangement between the ruling Workers’ Party and the nascent opposition.^123^ This altered many provisions of the 1952 Constitution but did not replace it. The amendments made in 1989 were buttressed in 1992 by another set of interim constitutional amendments, the transitional, time-limited ‘Small Constitution’, which was expected to be superseded in the future.^124^ Poland’s 1992 Small Constitution was, as Grover notes, ‘expressly temporary’ and stipulated that it was in force ‘pending the passing of a new Constitution’ or ‘until the adoption of a constitution’.^125^ The Small Constitution did not replace the 1952 Constitution outright, but after its five-year period it was duly replaced by the new 1997 Polish Constitution, helping to shepherd the country towards democracy. The operation of the Small Constitution provided a ‘test’^126^ of how the reformed state would work in practice. Meanwhile, comprehensive work was carried out in the legislature to decide the parameters of the new constitution.^127^ This allowed the legal transition to post-Communism to take place without substantial legal rupture.^128^ Poland also saw the restoration of its prewar presidential system, which was restored in 1989, with the form being confirmed in 1990.^129^ Like Russia, the model that Poland adopted in 1989 was influenced by the French Fifth Republic.^130^ However, unlike in Russia, this was treated as a restoration of continuity from the pre-Communist period, whereas in Russia it was truly novel. Furthermore, Poland became truly semi-presidential, and the position of president in Poland did not gain the immense ‘Crown-Presidential’^131^ powers that the Russian president did. Partlett could have also engaged with the South African Constitution, which is an effective example of a ‘pacted transition’ from authoritarianism to democracy that underwent an interim period of ‘consocialism’^132^ that combined elements of the old and new systems until being fully replaced. Nonetheless, there are also potential problems with interim constitutions, especially in authoritarian states: as Wu has recently argued, in developing countries such as Libya, Iraq and South Sudan, nominally interim constitutions have simply been left in place for decades, contributing to authoritarian practices and impeding democratisation.^133^

Here, the normative aspect comes into play. Although the Solidarity movement in Poland frayed into different factions post-independence, there was a consensus at the time of independence that the Communist regime needed to be removed, and a liberal democratic system established in its place. Such a normative consensus would be far more difficult to establish in Russia. Explanations of durable constitutional arrangements that fall back on political culture may hold up in certain contexts, but they offer fewer lessons in the case of countries with less democratic tradition such as Russia.^134^ A comparison between Poland and Hungary, which underwent very different transitions away from Communism, would have benefited the book. Hungary also faced many similar crises of legitimacy when it gained independence, but it took a separate road from Poland. Whereas Poland enacted the Small Constitution in 1992, in Hungary the country enacted a quickly drafted constitution in 1989 and continued to operate on that basis until 2011, creating a crisis of legitimacy.^135^ However, the Hungarian example is hardly a positive one, as in 2011 the newly elected Fidesz government used its constitutional majority in parliament to push through a new constitution. Whereas what Daly calls ‘constitutional repair’^136^ of the existing constitution looks possible in Poland, the prospects are less optimistic in Hungary, and while Partlett writes that the Russian Constitution only need undergo quite minor revisions, given how embedded authoritarianism has become in Russia there may be a less hopeful outlook for ‘constitutional repair’ in the country. Whatever constitutional form a future Russia takes, in a country like Russia, with limited democratic tradition, the option of interim constitution(s) looks like a constructive approach in order to avoid the dramatic rupture that the Russian system underwent in 1993.

## 8. Conclusion

*Why the Russian Constitution Matters* invites us to take the Russian Constitution seriously, and in doing so consider the lessons that can be learned from its entrenchment of authoritarian power. Partlett’s book details how constitutions in authoritarian states have real significance and makes an important contribution to studies of constitutional law and authoritarianism. The book’s central idea of the constitutional dark arts gives a framework which can be usefully applied to other constitutions in both non-democracies and backsliding democracies. History tells us that there are reasons to be hopeful. Experiences as diverse as the French Fifth Republic, the United States and Japan demonstrate that democratic constitutions can emerge from conditions of crisis and from countries with limited democratic traditions.

Partlett’s book makes several recommendations: one is the separation of the management and guardian roles of the president in order to avoid the ‘Frankenstate’ that Scheppele contemplates. Furthermore, there must be an embrace of a broader notion of constitutionalism rather than one simply centred on rights and courts. This means rejecting the ‘anti-politics’ of the Russian Constitution and its populist, plebiscitary approach to democracy. The president must stand among the horizontal divisions of power but must not stand above them. One potential remedy is the adoption of interim constitutions—’constitutions as process’—rather than simply a leap into the unknown that a new constitutional order can contemplate. Partlett urges a normative as opposed to a purely legalistic commitment to democratic constitutionalism, which may be the most difficult recommendation to enshrine. It is one thing to say that one has a normative commitment to democratic values and quite another to commit to it. None of the suggestions Partlett makes are a cure-all for constitutional authoritarianism in and of themselves, but they may point the way forward yet in a world where many constitutions are born out of and continue to operate in a state of crisis.

A more difficult question to answer is how to remedy a constitutional order that has already broken down. Constitutional repair may be impossible in a state such as Russia, with a full constitutional reset required instead, although such a prospect looks unlikely in the absence of a complete turnover of power. The failures of Russia’s 1993 Constitution teach us that any future constitutional process in Russia must be consultative, encompass a broad section of society, have sufficient international support and, crucially, ensure the horizontal apportionment of power rather than elevating the president above other offices. A constitution that is based on abstract guarantees of rights and a strong court system is unlikely to achieve these goals, as a centralising president is likely to simply manipulate these rules to their own advantage. Consideration of all these factors is necessary in order to face the creation of a post-Putin constitutional order in Russia without simply regressing to the failed norms and institutions of what came before.

## Footnotes

1
*Why the Russian Constitution Matters* 3.

2 ibid 23.

3 ibid.

4 ibid 3.

5 ibid 23.

6 ibid 8.

7 NW Barber, ‘What Is Constitutional Ideology?’ (2024) 22 ICON 653, 654.

8 William Partlett, ‘Crown-Presidentialism’ (2022) 20 ICON 204.

9 William Partlett and Mikhail Krasnov, ‘Russia’s Non-transformative Constitutional Founding’ (2019) 15(4) EuConst 644, 664–7.

10 William Partlett, ‘Separation of Powers without Checks and Balances: The Failure of Semi-presidentialism and the Making of the Russian Constitutional System, 1991–1993’ in Tatiana Borisova and William B Simons (eds), *The Legal Dimension in Cold-War Interactions* (Brill Nijhoff 2012).

11 William Partlett, ‘Russia’s 2020 Constitutional Amendments: A Comparative Analysis’ (2021) 23 Cambridge Yearbook of European Legal Studies 311.

12
*Why the Russian Constitution Matters* 2.

13 Eric Barendt, ‘Is There a United Kingdom Constitution?’ (1997) 17 OJLS 137, 141.

14 Samantha Lomb, *Stalin’s Constitution: Soviet Participatory Politics and the Discussion of the 1936 Draft Constitution* (Taylor & Francis 2017).

15 Neil J Diamant, *Useful Bullshit: Constitutions in Chinese Politics and Society* (Cornell UP 2022).

16 Melissa Crouch, *The Constitution of Myanmar: A Contextual* Analysis (Bloomsbury 2019).

17 Eugénie Mérieau, *Constitutional Bricolage: Thailand’s Sacred Monarchy vs. the Rule of Law* (Hart Publishing 2022).

18 Maria Smirnova and Chris Thornhill, ‘A Sociological Approach to the Russian Constitution’ (2016) 15 Comparative Sociology 747, 748–9.

19 Tom Ginsburg and Richard R Beeman, ‘Beyond Window Dressing: Constitutions in Authoritarian Regimes’ in Rogers M Smith and Richard R Beeman (eds), *Modern Constitutions* (University of Pennsylvania Press 2020) 133.

20 David Landau, ‘Abusive Constitutionalism’ (2013) 47 UC Davis L Rev 189, 212.

21 Henry E Hale, ‘The Informal Politics of Formal Constitutions: Rethinking the Effects of “Presidentialism” and “Parliamentarism” in the Cases of Kyrgyzstan, Moldova, and Ukraine*’ in Tom Ginsburg and Alberto Simper (eds), *Constitutions in Authoritarian Regimes* (CUP 2013) 240.

22 William E Pomeranz, ‘Putin’s 2020 Constitutional Amendments: What Changed? What Remained the Same?’ (2021) 6 Russian Politics 6.

23 Grigoriy Golosov, *Authoritarian Party Systems: Party Politics in Autocratic Regimes, 1945–2019* (World Scientific 2022).

24 See ch 3 of *Why the Russian Constitution Matters*.

25 Laurence Claus, ‘The Law of Constitutional Capture’ (2025) 19 ICL Journal 267.

26 Moshe Haspel, Thomas F Remington and Steven S Smith, ‘Lawmaking and Decree Making in the Russian Federation: Time, Space, and Rules in Russian National Policymaking’ (2006) 22 Post-Soviet Affairs 249, 257.

27 A senior Yeltsin advisor is quoted as using this term. ibid 253.

28 The rapid pace of law making in the Russian State Duma is such that it has been referred to as a ‘mad printer’. See Julian G Waller, ‘Mimicking the Mad Printer: Legislating Illiberalism in Post-Soviet Eurasia’ (2023) 70 Problems of Post-Communism 225, 230; Olga Romanova, ‘Legislation as a Tool for Expanding the Power of the State and Limiting Political and Civil Rights in Modern Russia’ (Istituto Affari Internazionali 2022) <www.iai.it/it/pubblicazioni/c03/legislation-tool-expanding-power-state-and-limiting-political-and-civil-rights> accessed 3 March 2026.

29
*Why the Russian Constitution Matters* 97.

30 Fabian Burkhardt, ‘Institutionalizing Personalism: The Russian Presidency after Constitutional Changes’ (2021) 6 Russian Politics 50.

31
*Why the Russian Constitution Matters* 16.

32 ibid 15.

33 ibid 62-63.

34 ibid 19.

35 ibid 34.

36 ibid 39.

37 Michael McFaul, ‘Choosing Autocracy: Actors, Institutions, and Revolution in the Erosion of Russian Democracy’ (2018) 50 Comparative Politics 305, 312; Jody LaPorte, ‘Russia’s Superpresidency’ in Susanne A Wengle (ed), *Russian Politics Today* (CUP 2023); Haspel, Remington and Smith (n 26) 250.

38 eg Timothy Frye, *Weak Strongman: The Limits of Power in Putin’s Russia* (Princeton UP 2021); Tony Wood, *Russia without Putin: Money, Power and the Myths of the New Cold* War (Verso 2020).

39
*Why the Russian Constitution Matters* 71.

40 ibid 62.

41 ibid 54.

42 ibid 75.

43 ibid 95.

44 ibid 117.

45 ibid 124, 127, .

46 This has become explicit since the 2020 constitutional amendments, see Pomeranz (n 22) 6; Partlett, ‘Russia’s 2020 Constitutional Amendments’ (n 11) 326.

47 eg Victoria Schwartz, ‘The Influences of the West on the 1993 Russian Constitution’ (2009) 32(1) Hastings Int’l & Comp L Rev 101, 124–5; Oxana Gaman-Golutvina, ‘Parliamentary Representation and MPs in Russia: Historical Retrospective and Comparative Perspective’ in Elena Semenova, Michael Edinger and Heinrich Best (eds), *Parliamentary Elites in Central and Eastern Europe* (Routledge 2014) 243; Timothy J Colton and Cindy Skach, ‘A Fresh Look at Semipresidentialism: The Russian Predicament’ (2005) 16 Journal of Democracy 113.

48
*Why the Russian Constitution Matters* 25.

49 ibid.

50
*Why the Russian Constitution Matters* 18.

51 ibid 19.

52 ibid 22.

53 ibid 38.

54 Lomb (n 14).

55 Bui Ngoc Son, ‘Russia’s Big-Bang Constitutional Amendments’ (2020) 53 NYUJ Int’l Law & Pol 751.

56
*Why the Russian Constitution Matters* 143.

57 ibid 144–5. Indeed, prominent Putin advisor and onetime ‘grey cardinal’ Vladislav Surkov stated that the reforms were necessitated by the ‘hyper-presidential’ tendencies within the extant Russian Constitution. RIA Novosti, ‘Politologi otsenili mnenie Surkova o giperprezidentskoi sisteme’ [Political Scientists Assessed the Opinion of Surkov about the Hyperpresidential System] *RIA Novosti* (Moscow, 26 February 2020) <https://ria.ru/20200226/1565224593.html> accessed 2 February 2026.

58 Derek S Hutcheson and Ian McAllister, ‘Consolidating the Putin Regime: The 2020 Referendum on Russia’s Constitutional Amendments’ (2021) 6 Russian Politics 355.

59 Barber (n 7).

60 Partlett, ‘Russia’s 2020 Constitutional Amendments’ (n 11) 321.

61 Tom Ginsburg and Alberto Simpser, ‘Introduction’ in Ginsburg and Simpser, *Constitutions in Authoritarian Regimes* (n 21) 2–8.

62 Gagerin Mitin, ‘Organ novoi vetvi vlasti’ [The Organ of a New Branch of Power] *Vedemosti* (Moscow, 20 November 2020) <www.vedomosti.ru/opinion/articles/2020/11/19/847664-organ-novoi> accessed 2 February 2026; Marina Aksenova and Iryna Marchuk, ‘Reinventing or Rediscovering International Law? The Russian Constitutional Court’s Uneasy Dialogue with the European Court of Human Rights’ (2018) 16 ICON 1322.

63 Giorgio Agamben, *State of Exception* (University of Chicago Press 2008) 10.

64
*Why the Russian Constitution Matters* 58.

65 The term comes from Stephen GF Hall and Thomas Ambrosio, ‘Authoritarian Learning: A Conceptual Overview’ (2017) 33 East European Politics 143.

66 Andrey Shirvindt, ‘Former Soviet States of Eastern Europe, Caucasus and Central Asia’ in Mathias Siems and Po Jen Yap (eds), *The Cambridge Handbook of Comparative Law* (CUP 2024).

67 Alex Mills and Daniel Joyce, ‘Non-governmental Organisations and International Norm Transmission on the Fault Lines of the International Order’ (2006) 19 Cambridge Review of International Affairs 11.

68 Sean P Roberts, ‘Converging Party Systems in Russia and Central Asia: A Case of Authoritarian Norm Diffusion?’ (2015) 48 Communist and Post-Communist Studies 147.

69 Waller uses the term ‘hegemonic diffusion’. Waller (n 28) 226.

70 Edward Lemon and Oleg Antonov, ‘Authoritarian Legal Harmonization in the Post-Soviet Space’ (2020) 27 Democratization 1221, 1228.

71 Waller (n 28) 232.

72 Petra Stykow, ‘The Devil in the Details: Constitutional Regime Types in Post-Soviet Eurasia’ (2019) 35 Post-Soviet Affairs 122, 132.

73
*Why the Russian Constitution Matters* 134.

74 Ilyas Saliba, ‘Change or Charade? Morocco’s Constitutional Reform Process 2011’ (2016) 57 Orient-Deutsche Zeitschrift für Politik, Wirtschaft und Kultur des Orients 52.

75 Mérieau (n 17).

76
*Why the Russian Constitution Matters* 183.

77 ibid 184.

78 For Partlett’s discussion of this, see ibid 187.

79 Norbert Kersting, Margarita Zavadskaya, and Tiphaine Magne, ‘Direct Democracy Integrity in Modern Authoritarian Systems’ (2023) 5 Politics of the Low Countries 90.

80 András Körösényi, Gábor Illés and Attila Gyulai, *The Orbán Regime: Plebiscitary Leader Democracy in the Making* (Routledge 2020) 84–5.

81
*Why the Russian Constitution Matters* 226.

82 ibid 195.

83 ibid 170.

84 ibid 195.

85 ibid 171.

86 Caroline Von Gall, ‘From “Living Constitutionalism” to “Zombie Apocalypse”. Valery Zorkin, the Constitutional Court and Russian Authoritarianism’ (2024) 50 Russian History 89.

87 Partlett, ‘Separation of Powers’ (n 10) 136.

88 Von Gall (n 86).

89
*Why the Russian Constitution Matters* 155.

90 Yulia Khalikova, ‘Constitutional Review and Dissenting Opinions in Nondemocracies: An Empirical Analysis of the Russian Constitutional Court, 1998–2018’ (2020) 21 Экономическая социология (Ekonomicheskaia sotsiologiia) 129.

91 ibid.

92 Zorkin offers a defence of ‘constitutional identity’ in terms of ‘restraining the excessive expansion of supranational regulation, using the powers of the Constitutional Court to achieve this goal, if necessary’. Valerii Zorkin, ‘Konstitutsionnaiia identichnost’ Rossii: doktrina i praktika’ [Constitutional Identity of Russia: Doctrine and Practice] (2017) 4(58) Zhurnal konstitutsionnogo pravosudiia 1.

93 TASS, ‘Zor’kin: popravki k konstitutsii pozvolili dostich’ balansa liberalizma i solidarizma’ [Zorkin: Amendments to the Constitution Will Achieve a Balance of Liberalism and Solidarism] *TASS* (Moscow, 13 December 2023) <https://tass.ru/politika/19518083> accessed 3 March 2026.

94
*Why the Russian Constitution Matters* 173.

95 ibid 17.

96 ibid 223.

97 Ginsburg and Beeman (n 19) 150.

98 Inder S Marwah, *Legalizing the Revolution: India and the Constitution of the Postcolony* (CUP 2025) 7.

99 Landau (n 20) 256.

100 Kim Lane Scheppele, ‘The Rule of Law and the Frankenstate: Why Governance Checklists Do Not Work’ (2013) 26 Governance 559.

101 Rosalind Dixon, ‘Constitutional Rights as Bribes’ (2018) 50 Conn L Rev 767.

102 Paul Chaisty and Stephen Whitefield, ‘Building Voting Coalitions in Electoral Authoritarian Regimes: A Case Study of the 2020 Constitutional Reform in Russia’ (2023) 39 Post-Soviet Affairs 273.

103 Claus (n 25) 26.

104 Anders Aslund, *How Capitalism Was Built: The Transformation of Central and Eastern Europe, Russia, the Caucasus, and Central Asia* (CUP 2013).

105
*Why the Russian Constitution Matters* 209.

106 For a glowing early overview of the Constitution’s commitments to international law, see Gennady M Danilenko, ‘The New Russian Constitution and International Law’ (1994) 88 AJIL 451.

107 European Commission for Democracy through Law (Venice Commission), ‘Draft Opinion on the Constitution of the Russian Federation as Adopted by Popular Vote on 12 December 1993’ (Council of Europe, 15 February 1994) <https://share.google/3q1mgmLCbgyQxPfi3> accessed 15 February 2026.

108 Aksenova and Marchuk (n 62).

109 Zotééva and Kragh call this an example of politics interfering with the Russian legal system and ‘a means to resolve the law and politics dilemma in Russia’. Anna Zotééva and Martin Kragh, ‘From Constitutional Identity to the Identity of the Constitution: Solving the Balance of Law and Politics in Russia’ (2021) 54 Communist and Post-Communist Studies 176, 183.

110
*Why the Russian Constitution Matters* 157, .

111 ibid 160.

112 ibid 166.

113 ibid 166-167.

114 ibid 176-177.

115 ibid 170.

116 For an overview, see Jennifer M Piscopo and Peter M Siavelis, ‘Chile’s Constitutional Chaos’ (2023) 34 Journal of Democracy 141.

117 The term comes from Andrew Arato, ‘Forms of Constitution Making and Theories of Democracy’ (1995) 17 Cardozo L Rev 191, 198.

118 Partlett, ‘Separation of Powers’ (n 10) 135.

119 Arnaud de Borchgrave, ‘Using Cues of the Past’ *Washington Times* (Washington, 10 May 2005) <http://csis-website-prod.s3.amazonaws.com/s3fs-public/legacy_files/files/media/csis/press/050510_de_borchgrave_twt.pdf> accessed 15 February 2026.

120 Leena Grover, ‘Out of the Shadows: Illuminating the Distinctiveness and Exceptional Use of Interim Constitutions’ (2024) 22(1) ICON 83.

121 Jacek Wojnicki, ‘The Issue of Restitution of Local Self-Governments in Poland and Croatia in the Late 20th Century—the Question of Their Constitutionalisation’ (2021) 30 Studia Iuridica Lublinensia 577, 581.

122 Wojnicki (n 119).

123 Grzegorz Górski, ‘Constitutional Changes in Poland between 1989 and 1997’ (2014) 1 Law and Administration in Post-Soviet Europe 5, 6–7.

124 Maria Kruk, ‘Stability and Changes in the Constitution of the Republic of Poland’ (2015) 9(2) Ius Novum 11, 12.

125 Grover (n 120) 98.

126 Kruk (n 124) 12.

127 ibid 12–13.

128 Grover (n 120) 85.

129 Jacek Wojnicki, ‘The Systemic Transformation in Poland—Determinants and Limitations’ in Artur Adamczyk, Goran Ilik and Kamil Zajackowski (eds), *Balkan Ambitions and Polish Inspirations* (Centre for Europe, University of Warsaw 2022) 99–100.

130 Wojnicki (n 129) 96.

131 Partlett, ‘Crown-Presidentialism’ (n 8).

132 Andrew Arato, ‘Interim Imposition’ (2004) 18 Ethics & International Affairs 25, 31.

133 Lorraine Wu, ‘Sham Interim Constitutions’ (2025) 1 Comparative Constitutional Studies 1 (published online ahead of print 2025) <https://doi.org/10.4337/ccs.2025.0055>.

134 Andrew Arato, ‘Constitution and Continuity in the East European Transitions—Part One: Continuity and Its Crisis’ (1993) 3 Tilburg Foreign Law Review 345, 350.

135 ibid 368.

136 Tom Gerald Daly, ‘Constitutional Repair: A Comparative Theory’ (2025) 73 Am J Comp L 1.

